# Draft Genome Sequence of a Clinical Strain of *Yersinia enterocolitica* (IP10393) of Bioserotype 4/O:3 from France

**DOI:** 10.1128/genomeA.00150-12

**Published:** 2013-02-21

**Authors:** Cyril Savin, Lionel Frangeul, Laurence Ma, Christiane Bouchier, Ivan Moszer, Elisabeth Carniel

**Affiliations:** a Yersinia Research Unit, National Reference Laboratory, Institut Pasteur, Paris, France; b Genome Bioinformatics Platform, Institut Pasteur, Paris, France; c Genomic Platform, Institut Pasteur, Paris, France

## Abstract

We sequenced the genome of a clinical isolate of *Yersinia enterocolitica* (IP10393) from France. This strain belongs to bioserotype 4/O:3, which is the most common pathogenic subgroup worldwide. The draft genome has a size of 4,463,212 bp and a G+C content of 47.0%, and it is predicted to contain 4,181 coding sequences.

## GENOME ANNOUNCEMENT

*Yersinia enterocolitica* is an enteric pathogen that usually causes fever, diarrhea, and abdominal pain in humans. Infections are often moderate and occur predominantly in young children. In elderly patients with underlying disorders (cirrhosis, iron overload, diabetes) severe systemic infections are not rare (1, 2).

*Y. enterocolitica* has a worldwide distribution but predominates in cold and temperate countries. It is the third bacterial cause of diarrhea in Europe after *Salmonella* and *Campylobacter* (3). The species is divided into six biotypes with various levels of pathogenicity: biotype 1B (highly pathogenic) has a limited geographic distribution (mostly the United States and Japan), while biotypes 1A (nonpathogenic) and 2 to 5 (moderate pathogenicity) are ubiquitous (2, 4). *Y. enterocolitica* is also divided into O-serotypes. The most widespread bioserotype worldwide is 4/O:3 (Europe, Canada, Japan, New Zealand) (3, 5–7). In the United States, biotype 1B strains predominated until the late 1980s but have now been replaced by 4/O:3 strains (5). Pigs are the main reservoir (2).

To date, only two genome sequences of *Y. enterocolitica* 4/O:3 strains are publically available: one clinical strain from Germany (8) and one swine isolate from The Philippines (9). The availability of more genome sequences of this common and widespread enteric pathogen would help in increasing our understanding of its diversity and facilitating the development of new molecular diagnostic tools.

Here we sequenced the genome of strain IP10393, a bioserotype 4/O:3 *Y. enterocolitica* strain isolated in France in 1982 from the stools of a patient presenting with diarrhea. Genome sequencing was performed using the Roche 454 GS-FLX titanium (Beckman Coulter Genomics). The run generated 750,117 reads corresponding to 226 Mb of chromosomal sequence, as strain IP10393 had lost its pYV plasmid upon subcultures. The mean read length was ∼300 bp. The data were assembled using Newbler assembler version 2.3 (Roche) into 101 contigs grouped into 8 scaffolds. Most of the gaps between the contigs were manually closed by Sanger sequencing of PCR products, yielding a final set of 12 contigs with a size ranging from 34,829 bp to 1,531,738 bp (N50 = 870,086 bp). The resulting draft genome had a size of 4,463,212 bp and a G+C content of 47.0%, which is similar to that of other *Y. enterocolitica* genomes (8–11). Genome annotation using Rapid Annotation using Subsystem Technology (RAST) (http://rast.nmpdr.org/) (12) predicted 4,181 coding sequences (CDS), 64 tRNA genes, and 5 rRNA genes. The average G+C content for each predicted CDS was 48.1%. Numerous copies of insertion sequences were present: 52 of IS*1667*, 8 of IS*sod5*, 7 of IS*1668*, and 3 of IS*Psy4*. The known virulence determinants *ail*, *inv*, and *yst* were also present.

The availability of this new chromosomal sequence of a *Y. enterocolitica* 4/O:3 isolate may help in the identification of the core genome of these strains and a variable pool that differentiates subgroups.

### Nucleotide sequence accession numbers. 

The IP10393 sequence has been deposited at DDBJ/EMBL/GenBank under the accession numbers CAOV01000001 to CAOV01000012 and BioProject accession number PRJNA177695. The version described in this paper is the first version.
